# Yellowing treatment transforms sensory profile of Qianlin cha (*Camellia cuspidata*): Key aroma compounds and quality enhancement

**DOI:** 10.1016/j.fochx.2026.104102

**Published:** 2026-06-18

**Authors:** Fei Ye, Xueping Wang, Xiaoyan Qiao, Anhui Gui, Panpan Liu, Lin Feng, Jin Teng, Jinjin Xue, Binghua Zhang, Pengcheng Zheng, Shiwei Gao

**Affiliations:** a Fruit and Tea Research Institute, Hubei Academy of Agricultural Sciences, Wuhan 430064, China; b Guangdong Provincial Key Laboratory of Tea Plant Resources Innovation and Utilization, Tea Research Institute, Guangdong Academy of Agricultural Sciences, Guangzhou 510610, China; c Danding Tea Co.,Ltd., Danjiangkou County, Shiyan 442717, China

**Keywords:** Qianlin cha, Yellowing, Physicochemical properties, Aroma compounds, ROAV, HS-SPME-GC–MS-O

## Abstract

*Qianlin cha* (QLC), a traditional Taoist herbal tea derived from *Camellia cuspidata*, is prized for its health benefits, but its bitter taste is obvious and has a herbal flavor. In order to reduce the bitterness in QLC, enhance the fragrance of florals, and develop new products. The purpose of this study is to reduce the bitterness and astringency of QLC and improve the fragrance of florals through the application of yellowing process. Comprehensive assessments, including sensory evaluation, color analysis, partial least squares discriminant analysis (PLS-DA) of physicochemical parameters, headspace solid-phase microextraction gas chromatography–mass spectrometry (HS-SPME-GC–MS), and gas chromatography–olfactometry (GC-O), were employed to identify key determinants of QLC's sensory appeal. The yellowing-treated QLC (QLC-Y) exhibited significantly improved sensory scores, marked color transformation toward desirable yellow hues with reduced *a* and *b* values, and enhanced levels of amino acids and soluble sugars (*P* < 0.05), contributing to a sweeter taste profile. In contrast, bitter and astringent ester catechins decreased notably, indicating superior physicochemical characteristics. Aroma analysis indicated the elevated aroma indices (*P* < 0.05), leading to the identification of four aroma-active compounds by GC-O: linalool, nonanal, sulcatone, and (*E*,*E*)-2,4-heptadienal. These results indicated that the yellowing treatment alters the sensory profile of QLC from herbaceous and bitter notes to a sweeter, more floral character by modifying its volatile compounds. This study provides preliminary mechanistic evidence suggesting that yellowing treatment associated with improvements in *Camellia cuspidata* tea quality, which could offer a scientific basis for premium herbal tea production.

## 1 Introduction

*Camellia sinensis* (tea) is the world's most consumed aromatic beverage after water, valued for its sensory appeal and health benefits (Guo et al., 2019). Growing recognition of tea's functional properties has spurred interest in alternative botanical infusions. Examples include jasmine tea (*Jasminum sambac*), which has documented neuroactive and antioxidant effects (An et al., 2023; An et al., 2022; Chen & Li, 2025) and whose traditional utilization in China is described (Yang et al., 2012); vine tea from *Ampelopsis grossedentata*, noted for its distinctive aroma and health benefits (Li, Qianqian, et al., 2024); Jiaogulan tea from *Gynostemma pentaphyllum*, widely used as an herbal tea, vegetable, functional food, and potential natural sweetener (Zhang et al., 2022); hawk tea from *Litsea coreana* Lévl. var. lanuginose, appreciated for its unique flavor and potential health effects (Bao et al., 2023); and kuding tea from *Ilex latifolia* or *Ligustrum robustum*, popular in southern China and associated with anti-inflammatory and cardiovascular benefits (Eigel et al., 2015; Li et al., 2013; Ping et al., 2014). Many plant resources have thus been transformed into regional specialty teas with distinctive tastes or physiological activities (Wang et al., 2016).

Qianlin cha (QLC), a traditional Taoist beverage processed from *Camellia cuspidata*, has been consumed in Hubei province for centuries and was once offered as a tribute tea during the Ming dynasty. Traditionally, QLC is credited with anti-inflammatory, bactericidal, hypoglycemic, and hypolipidemic effects. Despite these uses, QLC processing currently follows the green-tea model developed for *C. sinensis*, even though QLC's sensory and chemical characteristics differ markedly from green tea. QLC leaves are typically greenish-yellow with a stout appearance, and their infusion yields a bright golden liquor with a pronounced green/herbaceous aroma and a strongly bitter, astringent taste; the brewed infusion is described as bright yellow, full-bodied, and tender. By contrast, the favorable flavors and health attributes of green tea have been attributed to constituents such as tea polyphenols, amino acids, and caffeine (Hu et al., 2022), as well as to specific odorants, including ethylbenzene and heptanal (Zhu et al., 2018). The traditional processing of Qianlin tea - natural spreading, fixation and drying - is completely in accordance with the green tea process. However, there is still a lack of systematic research on how processing parameters affect the formation of aroma compounds. Nevertheless, systematic studies on the principal biochemical and aromatic constituents of QLC remain scarce (Zhang, Zhang, et al., 2024), and no tailored processing technologies have been established to optimize QLC flavor.

Yellowing is a post-fixation treatment that can markedly improve tea quality by developing sweeter, mellower flavor; it consists of piling fixed or partially dried leaves in a warm, humid environment for a defined period (Wei et al., 2021, Wei et al., 2023; Ye et al., 2022). During yellowing, gallated catechins and some glycosides are progressively degraded, while the free amino acid content rises, resulting in changes that soften bitterness and astringency. Concurrently, the abundance of volatiles associated with sweet and floral notes (for example, 3-methylbutyric acid) increases, shifting the aroma toward floral, sweet, and fruity profiles (Huang et al., 2023). Overall, yellowing has been shown to improve sensory quality, chemical composition, and aroma in several tea types. We assume that the yellowing process will induce a unique set of chemical and sensory changes in the tea processing, which may be different from the results of yellowing in tea leaves, which may lead to the formation of characteristic aroma-active compounds of Qianlin Cha. However, yellowing has not yet been applied to QLC, and the detailed transformation dynamics of its physicochemical and volatile constituents remain unexplored.

To address this gap, the present study compares traditionally processed QLC and yellowing-processed QLC, tracking dynamic changes in sensory attributes, color, physicochemical composition, and aroma compounds. We combined sensory evaluation and colorimetric analysis with physicochemical profiling, HS-SPME–GC–MS, relative odor activity value (ROAV) calculations, gas chromatography–olfactometry (GC-O), and absolute quantification to identify the compounds that most strongly influence QLC acceptability. These results could identify potential yellowing-induced metabolic markers specific to *Camellia cuspidata*, and define the key volatile and non-volatile compounds contributing to the characteristic flavor profile of Qianlin Cha, which to provide a mechanistic foundation for optimizing, mechanizing, and standardizing Qianlin Cha production.

## 2 Materials and methods

### 2.1 Preparation of QLC samples

QLC samples were manufactured by an experienced tea worker (Binghua Zhang) at Danding Tea Co., Ltd. (Danjiangkou County, Hubei, China). More than 100 kg of one-bud shoots of the cultivar *Camellia cuspidata* Wright var. *cuspidata* were harvested in the Shiyan area on April 10, 2023. Harvested material was processed into two product types (green and yellow) following the procedures below. After spreading, leaves destined for both treatments underwent fixation and cooling; thereafter, the batches were split and finished either by direct drying (green tea procedure) or by yellowing followed by drying (yellow tea procedure). Spreading was performed for 6–8 h at a layer thickness of 30–50 mm until leaf moisture reached 68–70% (*w*/w). Fixation was carried out using a hot-roller machine (model 6CST-80, Zhejiang Shang Yang Co., Ltd., China) for 2.5 min, with roller temperatures of 240, 220, and 200 °C, a roller speed of 24 rpm, and an indicated fixation duration of 72–75 s. Fixed leaves were cooled and softened on mesh using a moisture-regaining device (YJY-20, Ningbo Yaojiangyuan Machinery Co., Ltd.). For the green-tea workflow (QLC-G), leaves were dried directly in a box hot-air dryer (JY-6CHZ-7B, Fujian Jiayou Machinery Co., Ltd., Fujian, China) at 70 °C for 2 h. For the yellowing treatment (QLC-Y), the fixed leaves were wrapped in a dampened cotton cloth and placed in a constant temperature and humidity room. The process parameters (28–30 °C, 85–90% relative humidity, 24 h) were selected based on established local production practices. Subsequently, the leaves were dried under the same conditions as QLC-G. Upon completion of manufacture, QLC-G and QLC-Y were collected separately, subjected to immediate sensory assessment where feasible, and stored at −20 °C until further analysis. Each treatment was prepared and analyzed in triplicate.

### 2.2 Sensory quality assessment

The sensory evaluation was carried out by five expert tea tasters from the Institute of Fruit and Tea, Hubei Academy of Agricultural Sciences, following the Chinese national standards “Methodology for Sensory Evaluation of Tea” (GB/T 23776–2018) and “Tea Vocabulary” (GB/T 14487–2017). For each test, 3.0 g of sample was infused in 150 mL of freshly boiled distilled water for 4 min. The infusion was presented in white porcelain bowls and coded with randomized, blind identifiers. Each sample was evaluated in triplicate, and the entire blind evaluation procedure was repeated three times. Panelists (aged 29–46) assessed aroma and taste using smell and sip, with a 30-s interval between samples (Wu et al., 2022; Ye et al., 2022). Prior to formal scoring, panelists were familiarized with reference samples of graded intensity for each attribute, and then performed individual assessments using the prescribed intensity scales. Quantitative descriptive analysis (QDA) was conducted by five experts (3 male and 2 female), and the scores of five taste attributes (umami, sweetness, kokumi, bitterness, and astringency) were assessed on a ten-point scale, in which 0–2 represented “extremely weak”, 2–4 represented “weak”, 4–6 represented “neutral”, 6–8 represented “strong”, and 8–10 represented “extremely strong” (Xue et al., 2023).

In accordance with the guidelines of the Hubei Academy of Agricultural Sciences and institutional practices, formal ethical approval was not required for this sensory evaluation study, as it involved a low risk assessment of food products with healthy adult volunteers. However, the study was performed in compliance with ethical standards to protect participants' rights and privacy. All procedures adhered to the protocols for data protection and anonymity. All five expert tea tasters were fully informed about the study's objectives and procedures, and their informed consent was obtained prior to their participation.

### 2.3 Determination of QLC color quality

The color quality of QLC was evaluated using the CIELAB parameters *L*, *a*, and *b* in a three-dimensional color space. The *L* value represents brightness, ranging from 0 (pure black) to 100 (pure white). The *a* value spans from green (negative) to red (positive), while the *b* value ranges from blue (negative) to yellow (positive). Both dry tea leaves and brewed tea infusions were analyzed for color characteristics using a spectrophotometer (CM-5, Konica Minolta Investment Ltd., Shanghai, China), with distilled water serving as the baseline control (Cao et al., 2021; Ye et al., 2022). Higher *L* values indicate greater lightness, and the magnitudes of *a* and *b* are positively correlated with their respective chromatic hues (Li, Chaoyang, et al., 2024).

### 2.4 Determination of QLC physicochemical quality

Moisture content was measured using the 120 °C drying method (GB/T 8304–2013). Amino acid content was determined via the ninhydrin colorimetric method (GB/T 5009.124–2003). Total tea polyphenols were quantified using the iron tartrate colorimetric method (ISO 14502-1:2005), and soluble sugars were measured by the anthrone colorimetric method (NY/T 3030–2016).

Catechin analysis was performed by grinding tea leaves into a powder using a ball mill (MM400, Retsch, Haan, Germany) with zirconia beads for 1.5 min at 30 Hz. Tea powder (1.0 g) was extracted with 25 mL of 70% methanol in a 50 mL tube, vortexed for 30 s, and incubated in a 70 °C water bath for 10 min. After centrifugation at 4 °C for 10 min, the supernatant was collected. The extraction was repeated, and combined supernatants were filtered through a 0.22 μm microporous membrane for HPLC analysis (Waters 2695, USA) using the external standard method.

HPLC separation was conducted on an Agilent ZORBAX SB-Aq C18 column (4.6 mm × 250 mm, 5 μm) with a mobile phase consisting of solvent A (2% acetic acid in water) and solvent B (acetonitrile). The gradient program was as follows: 0–16 min, 6.5%–85% B; 16–25 min, 85%–75% B; 25–30 min, 75%–6.5% B; and 30–40 min, 6.5% B. Column temperature was maintained at 40 °C, injection volume was 10 μL, and flow rate was 1.0 mL/min (Ye et al., 2022). All measurements were performed in triplicate.

### 2.5 Analysis of volatile compounds by gas chromatography–mass spectrometry (GC–MS)

Volatile compounds were analyzed by headspace solid-phase microextraction (HS-SPME; Supelco, Bellefonte, PA, USA) coupled with GC–MS (Agilent 7890 A GC, 5975C MSD) following (Ye et al., 2022). The SPME fiber was initially conditioned according to the manufacturer's instructions before the first use. Prior to each subsequent analysis, the fiber was briefly reconditioned (e.g., at 250 °C for 10 min) in the GC injector to eliminate any potential carryover.

For analysis, the 3.0 g of dried tea leaves were placed in a 100 mL vial 20 μL of ethyl caprate (0.862 μg/mL) as the internal standard. The vial was equilibrated in a 50 °C water bath for 10 min, after which the SPME fiber was exposed to the headspace for 50 min before desorption at 240 °C for 3 min. Separation was performed on a DB-5MS capillary column (30 m × 0.25 mm × 0.32 μm).

The oven temperature was programmed as follows: 50 °C for 5 min, ramped to 180 °C at 3 °C/min (held for 2 min), then to 250 °C at 10 °C/min (held for 3 min). Helium was used as the carrier gas at a flow rate of 1.0 mL/min. MS detection employed electron impact ionization (70 eV) with a scanning range of 35–400 AMU. Compounds were identified by comparison with the NIST 20 library and linear retention indices (RIs) determined using C7–C40 alkanes as standards (Ye et al., 2022). Only compounds with a mass spectral matching score ≥ 700 were considered. RIs were calculated from published retention times. Relative volatile compound contents were normalized to the ethyl caprate internal standard. Terpene index = [Linalool] + Σ[Linalool Oxides] / ([Linalool] + Σ[Linalool Oxides] + [Geraniol])(Takeo, 1981).

### 2.6 ROAV calculation

Relative odor activity values (ROAVs) were calculated as the ratio of a compound's concentration to its odor detection threshold (ODT) (Liu et al., 2022). Volatile compound concentrations were obtained using: volatile compounds concentration = (volatile compounds peak area / ethyl caprate peak area) × ethyl caprate concentration (μg/L), ODT values were sourced from literature. Compounds with ROAV ≥1 were considered potential aroma contributors, and those with ROAV >10 were identified as major contributors to the tea's aroma profile (Schieberle & Peter, 1995).

### 2.7 GC–O analysis

GC–O was conducted following the GC–MS method in Section 2.5 with slight modifications. The aroma extract was split equally between the Olfactory Detection Port (ODP3, Gerstel, Germany). The MS Injector and transfer line temperatures were set at 230 °C and 260 °C, respectively, with 99.99% pure nitrogen as the carrier gas.

Five trained assessors from the Fruit and Tea Research Institute, Hubei Academy of Agricultural Sciences (Section 2.2), performed the GC–O evaluations. Each sample was analyzed twice, and aroma intensities (AI) were scored on a four-point scale: 1 = weak, 2 = moderate, 3 = strong, and 4 = extremely strong (Wang et al., 2020).

### 2.8 Aroma recombination experiments

The aroma recombination methods were modified from other research (Ma, Gao, Hu, et al., 2022; Zhu et al., 2018). For the aroma recombination experiment, the key floral compounds were added to the preheated non-floral matrix (60 °C water bath for 30 min) according to the actual content of the QLC sample. The mixture was immediately sealed and placed in a 60 °C water bath for 10 min to create a recombinant model.

The recombinant sample was evaluated by panelists according to the method of “Section 2.2 and Section 2.7”, who were trained intensively for precise recognition of different aroma attributes for two weeks (at least 30 h). Specific standards of different concentrations were diluted with 10% ethyl alcohol for training of sensory evaluation. These included nonanal representing a “sweet” aroma; linalool representing a “floral” aroma; hexanal representing a “grass” aroma; β-ionone representing a “woody” aroma, and (3Z)-hex-3-en-1-yl acetate representing a “roast” aroma.

### 2.9 Data analysis

All results were expressed as the mean of three replicates. Statistical significance was determined by one-way ANOVA followed by Duncan's multiple range test or Student's *t*-test, the data are presented as mean ± standard deviation (*n* = 3). Mean values with the different lowercase letters indicate significant difference with least significant difference (*P* < 0.05). Figures were generated using Origin 8.0 (Demo version, Northampton, MA, USA), and additional graphs were prepared in GraphPad Prism v8.0.2.263 (GraphPad Software Inc., San Diego, CA, USA). Partial least squares discriminant analysis (PLS-DA) for metabolite profiling was performed using MetaboAnalyst 6.0 (https://www.metaboanalyst.ca/MetaboAnalyst/faces/home.xhtml).

## 3 Results and discussion

### 3.1 Effect of yellowing treatment on QLC sensory qualities

**Fig. 1:**
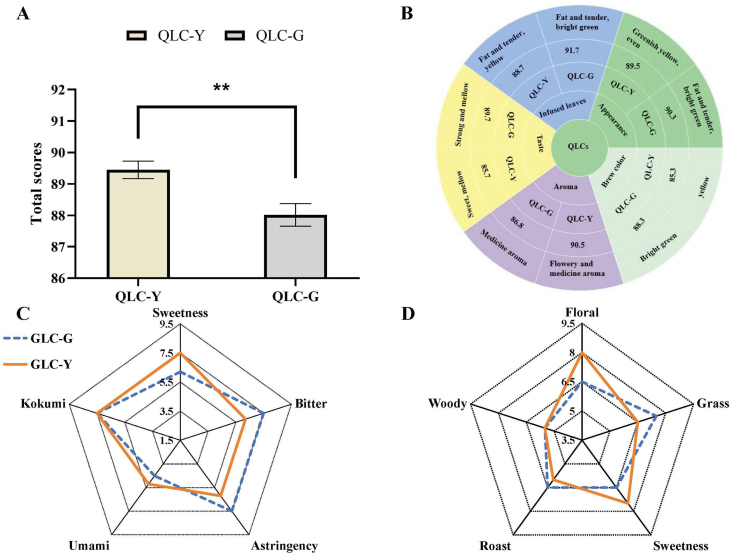
Sensory quality scores of Qinlin cha processed by different methods. (A) Total scores of Qinlin cha samples. (B) Vocabulary and scores for appearance, brew color, aroma, taste**,** and infused leaves of QLCs. (C) Quantitative descriptive analysis of taste attributes of Qinlin cha samples. (D) Quantitative descriptive analysis of aroma attributes of Qinlin cha samples. ** in bar charts indicate significant differences between samples (*P* < 0.01, *t*-test). QLC-Y, Qinlin cha processed by the yellow tea process; QLC-G, Qinlin cha processed by the green tea process. (For interpretation of the references to color in this figure legend, the reader is referred to the web version of this article.)

The sensory evaluation results for QLC-G and QLC-Y were shown in Fig. 1. The yellowing treatment markedly altered the sensory profile of the brew, specifically its color, aroma, and taste. Overall, QLC-Y achieved the higher total sensory score (90.75; Fig. 1A) and was described by panelists as having greenish-yellow dry leaves, a yellow infusion, a fresh yet mellow taste, a flowery aroma, and fat, tender infused leaves (Fig. 1B). QLC-Y received the highest aroma score (92.5), characterized as floral with medicinal notes, and a higher taste score (89.7), described as sweet and mellow; both aroma and taste scores were significantly greater than those of QLC-G (*P* < 0.05). In contrast, QLC-G scored higher for dry tea appearance, brew color, and infused-leaf appearance. We speculated the yellowing treatment could promoted release of alcohol-type volatiles and enhanced Strecker and oxidative degradation pathways, which likely contributed to these sensory shifts (Dong et al., 2023; Huang et al., 2023).

QDA of taste attributes showed that sweetness and umami intensities followed the order QLC-Y > QLC-G, whereas bitterness and astringency were greater in QLC-G; bitterness and astringency scores for QLC-G were 7.5 and 7.5, respectively (Fig. 1C). Kokumi intensity did not differ significantly between treatments (*P* > 0.05). As shown in Fig. 1D, floral and grassy notes dominated the aroma profile, with floral and grass descriptors scoring >6 on a 10-point intensity scale; woody notes remained low (≈5). The yellowing treatment led to a notable enhancement in floral and sweet flavors, alongside a simultaneous decline in grassy and roasted notes. These observations were consistent with earlier reports that yellowing substantially modulates umami, bitterness, and astringency in tea, reinforcing the role of the yellowing step in producing sweet, floral, and stable high-quality teas (Wei et al., 2021).

### 3.2 Effect of yellowing treatment on QLC color qualities

**Fig. 2:**
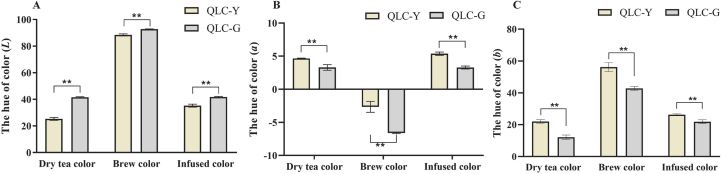
Color parameters of Qinlin cha processed by different methods. (A) Lightness (*L*) of QLCs. (B) Chromaticity *a* of QLCs. (C) Chromaticity *b* of QLCs. ** in bar charts indicate significant differences between samples (*P* < 0.01, *t*-test). QLC-Y, Qinlin cha processed by the yellow tea process; QLC-G, Qinlin cha processed by the green tea process. (For interpretation of the references to color in this figure legend, the reader is referred to the web version of this article.)

Color parameters for QLCs processed by different methods are presented in Fig. 2. The yellowing treatment produced clear changes in *L*, *a*, and *b* values across dry tea, brew, and infused leaves. QLC-G exhibited significantly higher *L* values (dry tea, brew, and infused leaves), indicating greater lightness compared with QLC-Y. By contrast, chromatic shifts in *a* and *b* associated with the yellowing treatment produced a noticeably deeper, more intensely colored infusion in QLC-Y, consistent with sensory panel observations. These color changes likely reflect ongoing chemical reactions during yellowing—enzymatic browning, pigment oxidation, polyphenol oxidation, isomerization, starch hydrolysis, and protein decomposition—that alter pigment composition and other constituents (Kręcisz et al., 2023; Peng et al., 2024).

### 3.3 Effects of yellowing treatment on the physicochemical qualities of QLCs

**Fig. 3:**
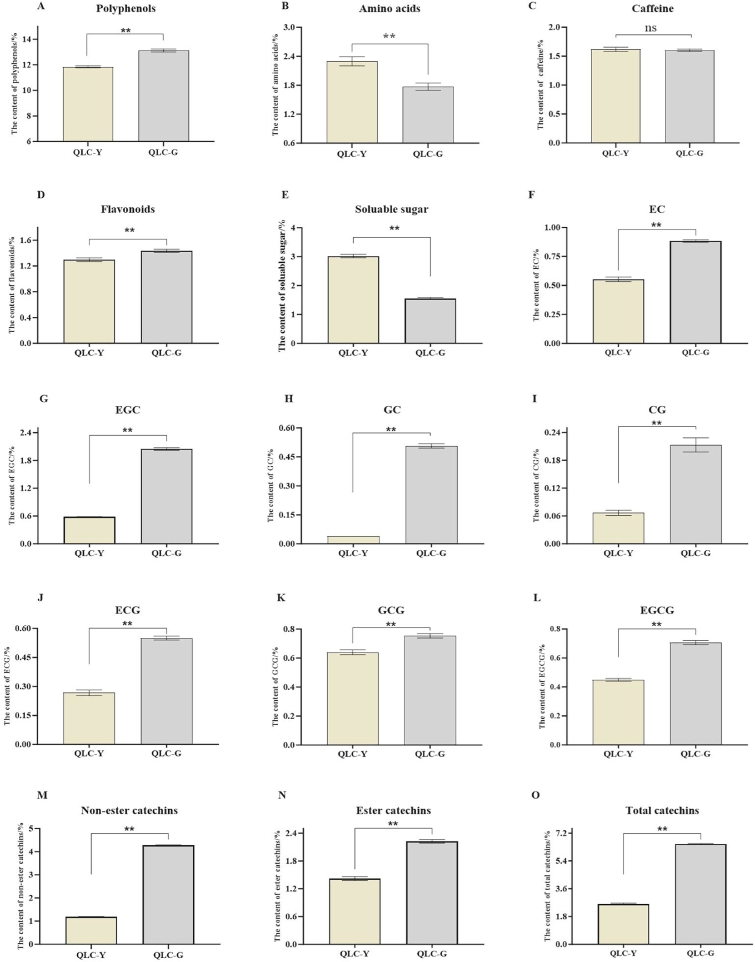
Difference analysis of non-volatile compounds. (A) Tea polyphenol; (B) Amino acids; (C) Caffeine; (D) Flavonoids; (E) Soluble sugar; (F) EC, epicatechin; (G) EGC, epigallocatechin; (H) GC, gallocatechin; (I) CG, catechin gallate; (J) ECG, epicatechin gallate; (K) GCG, gallocatechin gallate; (L) EGCG, epigallocatechin gallate; (M) Non-ester catechins; (N) Ester catechins; (O) Total catechins.

The chemical composition of QLCs is presented in Fig. 3. Specifically, polyphenols, amino acids, soluble sugars, caffeine, flavonoids, and catechins, including EC, GC, ECG, CG, EGC, GCG, and EGCG, were identified and quantified.

Amino acid and soluble sugar contents were significantly higher in QLC-Y samples (*P* < 0.01), whereas polyphenols, catechins (EC, GC, ECG, CG, EGC, GCG, EGCG, non-ester catechins, ester catechins, total catechins), and flavonoids were significantly higher in QLC-G samples (*P* < 0.01). Caffeine content remained stable across treatments (*P* > 0.05). Amino acids and sugars are key taste compounds, contributing to the umami and sweetness of tea infusions (Mao et al., 2018; Zhang, 2019). The elevated levels of amino acids and soluble sugars in QLC-Y correspond to a more distinct umami and sweet taste profile, which aligns with the sensory evaluation results for yellow-treated QLCs. Conversely, polyphenols—particularly catechins—are major contributors to tea bitterness and astringency (Jiang et al., 2022; Xu et al., 2018; Zhang, 2019). Concentrations of EC, GC, ECG, CG, EGC, GCG, and EGCG were significantly lower in QLC-Y, with non-ester catechins, ester catechins, and total catechins all higher in QLC-G.

Ester catechins, such as ECG, GCG, and EGCG, are considered the most biologically active tea components and the principal sources of bitterness and astringency (Wang et al., 2020). In this study, both ester catechins and total catechins were reduced in QLC-Y, suggesting lower bitterness and astringency intensity. The decrease in GC, C, EC, ECG, GCG, ester catechins, and total catechins in QLC-Y may be attributed to ester catechin degradation during yellowing (Fan et al., 2016; Guo et al., 2021). These findings demonstrate that the yellowing treatment modifies the physicochemical composition of QLCs, thereby affecting their taste and sensory quality.

### 3.4 Effects of yellowing treatment on the aroma and volatile compounds of QLCs

**Fig. 4:**
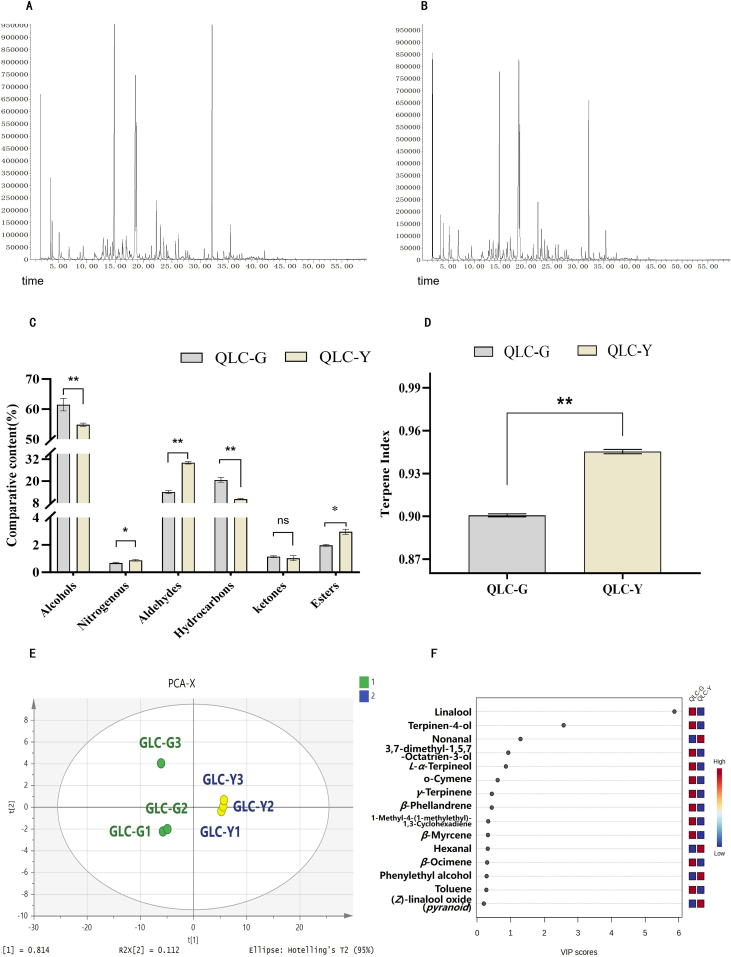
Analysis of differentially abundant volatile compounds (VCs) and aroma compounds between QLC-G and QLC-Y samples. (A-B). The chromatogram of QLC-G and QLC-Y. (C) Abundance of specific VOC categories in QLC-G and QLC-Y. (D) Terpene index. (E) Principal component (PC) 1 and PC2 values of the QLCs sample types. PCs were derived from partial least squares discriminant analysis (PLS-DA). (F) Top 15 aroma components with the highest VIP values.

Based on sensory and chemical analyses, QLC-Y and QLC-G samples were evaluated for volatile compounds (VCs) using HS-SPME-GC–MS. Consumers generally prefer floral aromas, and in total, 53 VCs were identified (Fig. 4C∼Fig. 4D), classified into six categories: alcohols, nitrogenous compounds, aldehydes, hydrocarbons, ketones, esters, and others. The highest content of volatile components in GLC samples was alcohols, accounting for more than 60% of the total volatile compounds (Fig. 4C), followed by aldehydes. After the yellowing treatment, the alcohols and hydrocarbons of the samples were significantly decreased (*P* < 0.05), and the nitrogen, alcohols and esters were significantly increased (*P* < 0.05), and the ketones content remained stable. Volatile compounds such as alcohols, aldehydes and ketones are important volatile components. The alcohols are mostly floral or sweet, aldehydes are often green or aromatic, ketones and esters are floral and fruity (Dong et al., 2023; Huang et al., 2023).

Alcohols were the most abundant VCs in both QLC-G and QLC-Y, accounting for 61.49% and 54.86% of total VCs, respectively, consistent with previous reports (Fan et al., 2019; L. Zhang, Zhang, et al., 2024). Alcohols are important aroma contributors in green tea; for instance, linalool imparts floral, fruity notes (Zhu et al., 2018), terpinen-4-ol has a strong floral aroma, and *α*-terpineol, (*E*)-linalool oxide (*furanoid*), and phenylethyl alcohol provide sweet and citrus notes (Wang et al., 2020; Zhou et al., 2022). In QLC-G, linalool, terpinen-4-ol, (*Z*)-linalool oxide (*pyranoid*), (*E*)-linalool oxide (*furanoid*), phenylethyl alcohol, and (*E*)-3-hexenol were dominant, representing 67.29%, 17.29%, 2.43%, 0%, 0.74%, and trace levels, respectively. After yellowing, levels of these alcohols (except linalool and terpinen-4-ol) increased, reaching 64.76%, 6.61%, 5.92%, 5.22%, 3.28%, and 2.96% in QLC-Y. The terpene index was significantly higher in QLC-Y (*P* < 0.01; Fig. 4B). Given that alcohols are key floral aroma contributors (Zhu et al., 2018), It is inferred that linalool, (*Z*)-linalool oxide (*pyranoid*), and benzyl alcohol contribute to the fresh, floral aroma profiles of QLC-Y.

A total of 14 aldehydes were identified. Prominent examples include nonanal with a rose fragrance (Xu et al., 2021), benzaldehyde was a almond odor (Xu et al., 2021), heptanal was nutty (Wang et al., 2023; Xu et al., 2023), hexanal was the smell of green/grassy, (*E,E*)-2,4-heptadienal was grassy/fresh (Wang et al., 2021), and octanal was green/grassy/fresh (Wang et al., 2020). QLC-Y exhibited significantly higher alkene contents (*P* < 0.01), approximately 30% greater than QLC-G, although heptanal, benzaldehyde, octanal, and (*E,E*)-2,4-heptadienal levels decreased, while hexanal and nonanal increased.

Sixteen hydrocarbons, five esters, and two ketones were detected. While hydrocarbons were previously considered minor contributors to tea aroma (Lin et al., 2019), recent studies report aromatic roles for *o*-cymene (Guo et al., 2021), *trans*-calamenene (herbal/spicy), 1,5,7-octatrien-3-ol was the smell of floral (Bao et al., 2023), *β*-myrcene (sweet), and enhanced the characteristic aroma profiles of the Dancong teas (Chung et al., 2020), including 3-methyl-tridecane (floral/fruity), (*E,E*)-3,5-octadien-2-one (green), *β*-phellandrene (terpenic/minty)(Hao et al., 2023), and toluene (paint/sweet)(Zhang, Zhang, et al., 2024). QLC-Y contained fewer hydrocarbons (Fig. 4A) and lower ketone content (*P* < 0.05), yet retained a strong floral-like aroma.

Esters such as methyl salicylate was a mint aroma (Cao et al., 2021), hexanoic acid 3-hexenyl ester was a flowery/fruity aroma (Xue et al., 2023), and dihydroactinidiolide (floral/rose-like)(Shao et al., 2022) were identified, along with ketones such as *β*-ionone (floral) (Wang, Ouyang, et al., 2022) and methylheptenone (characteristic aroma of fried mountain pepper oil). While the contents of these ketones varied stably, but the esters increased in QLC-Y (*P* < 0.05). The results indicated that the yellowing treatment effectively developed a prominent floral character in QLC-Y. It was hypothesized that the increase in esters, rather than ketones, were the key driver behind this aroma.

PLS-DA (Fig. 4C) was then performed and the cumulative contribution rate of the two principal components (PCs) was 99.7%, which means a majority of the information contained in the original data (Qiao et al., 2023). PLS-DA (R^2^ = 0.431, Q^2^ = -0.174) revealed a clear influence of yellowing process on QLCs at the biochemical level. These results were consistent with the sensory evaluations (Fig. 1D). The variable importance in projection factors (VIPs) derived from PLS-DA, which could be used to assess the importance of each variable; the higher VIP values, the greater contribution rates to the aroma profiles. There were 4 compounds showed the higher significance whose VIP is greater than 1, including the linalool, terpinen-4-ol, nonanal and 3,7-dimethyl-1,5,7-octatrien-3-ol, thus these components strongly influencing the aroma qualities of the QLCs samples (Fig. 3B), which maybe the relevant variables contributing to aroma flavor (Fig. 3D). VIP results (Fig. 4F) analysis showed that linalool, terpinen-4-ol, 3,7-dimethyl-1,5,7-Octatrien-3-ol and L-α-terpineol (which VIPs >1)were significantly decreased in QLC-Y. In contrast, the nonanal (which VIPs >1), phenylethyl alcohol, hexanal and (*Z*)-linalool oxide (*pyranoid*) (which VIPs <1) increased, which could be used to discriminate between the QLC-Y and QLC-G samples.

We speculated that during yellowing, gradual exterior-to-interior heat transfer promotes protein degradation and secondary biochemical reactions, which likely increased the concentrations of both linalool and nonanal, thereby intensifying floral and sweet notes, while the *(E,E)-2,4-*heptadienal contributes fatty, stale-like nuances. The yellowing treatment appears to suppress the impact of these latter two compounds, shifting the overall aroma balance toward the more desirable sweet-floral profile observed in QLC-Y.

### 3.5 Comparison of the relative odor activity values of key QLC odorants

The yellowing treatment induced substantial chemical transformations in aroma constituents, leading to notable shifts in the levels of volatile compounds during processing. Monitoring changes in the relative odor activity values (ROAVs) of key odorants was essential for elucidating the evolution of QLC aroma during yellowing. ROAV is widely applied to assess the contribution of volatile compounds to the overall aroma profile, with values exceeding 1 generally considered to make a perceptible impact.

In our analysis, 21 aroma components exhibited ROAVs greater than 1 (Table1). Among these, linalool characterized by sweet and floral characteristics, which had the showed the highest ROAV (350–649), This was followed by *β*-ionone—characterized by violet-like, floral, and raspberry-like notes—showed the second highest ROAV (447–490), the both aromas making the most significant contributors to the floral aroma. This was followed by (*E*)-2-octenal (fatty; ROAV = 410–506) and octanal (fatty; ROAV = 410–506). Collectively, the floral-like aroma profile of QLCs was primarily attributable to linalool, *β*-ionone, (*E*)-2-octenal, and octanal.

Other odorants with ROAVs >10 included nonanal (59–98), heptanal (11−12), octanal (71–144), (*E,E*)-2,4-heptadienal (31–44), hexanoic acid, geraniol (55–115), and methyl salicylate (refreshing; 12–30), etc. Notably, nonanal, n-Valeric acid *cis*-3-hexenyl ester, octanal and (*E*)-3-Hexen-1-ol increased significantly in QLC-Y samples, whereas compounds such as heptanal, (*E*,*E*)-2,4-heptadienal, L-α-terpineol, geraniol, and terpinen-4-ol decreased (*P* < 0.05). Odorants with ROAVs between 1 and 10 included (*E*)-linalool oxide (*furanoid*; fresh, floral; 1.41–1.69), hexanal (green, fresh; 3.76–7.00), (*E*)-*β*-ocimene (warm, foral, herbal, sweet; 0–3.28), terpinen-4-ol (pleasant, foral; 0.65–3.03), *γ*-terpinene (refreshing, lemon-like; 0.98–3.41), and 2-pentylfuran (beany, green beans; 5.17–6.40). The levels of (*E*)-linalool oxide (*furanoid*), hexanal, and methyl salicylate increased significantly in QLC-Y, whereas γ-terpinene, terpinen-4-ol, and 2-pentylfuran decreased (*P* < 0.05).

Many studies have shown that linalool and linalool oxide are the main aroma components (Dong et al., 2023). We speculated that the yellowing treatment changed the aroma composition, the grassy aroma changed into sweet and fruity aroma, and the oxidative degradation pathway was enhanced, which may be the reason for the decrease of linalool, while the linalool oxide increased.

### 3.6 Comparison of the main difference aroma compounds of QLC odorants

The aroma of QLC is a comprehensive reflection of its green, flower, sweet, baking, wood fragrance and other attributes. The aroma of QLC was not only closely related to its aroma components, but also affected by the synergistic or antagonistic effects between aroma compounds. Thus, elucidating the different aroma compounds and their interactions in QLC is essential for understanding the factors that affect its aroma quality.

**Fig. 5:**
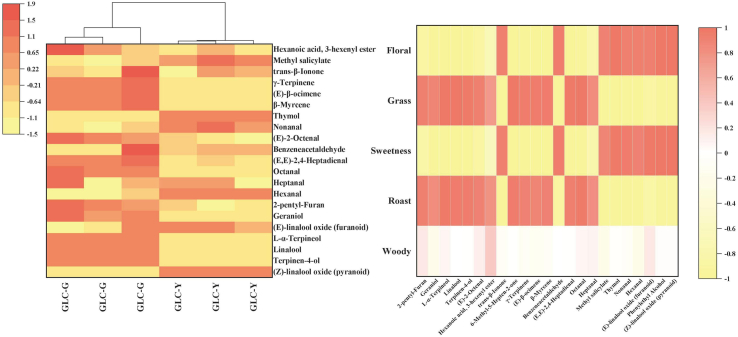
The heat map of main aroma components in QLCs and the correlation between aroma properties and main aroma compounds in QLCs. (A) Heatmap of 26 differential aroma components in QLC-G and QLC-Y; (B) Correlation between aroma attributes of QLCs and ROAV values, and their changes.

Pearson correlation coefficient was calculated to test the correlation between 26 different aroma components (ROVA >1) and five aroma attributes (green, sweetness, floral, baking and woody). 25 aroma compounds correlated closely with aroma attributes (*r* > 0.8), indicating that they are important factors underlying the aroma attributes of QLCs. The sweetness and floral attributes of QLCs were positively correlated with (*Z*)-linalool oxide (*pyranoid*), phenylethyl alcohol, hexanal, nonanal, methyl salicylate, benzeneacetaldehyde, β-ionone, while the heptanal, octanal, (*E*,*E*)-2,4-heptadienal, *o*-cymene, *β*-myrcene, (*E*)-β-ocimene, *γ*-terpinene, hexanoic acid, 3-hexenyl ester, (*E*)-2-octenal, terpinen-4-ol, linalool, L-*α*-terpineol, geraniol, 2-pentyl-furan (Fig. 5).

### 3.7 Comparison of aroma-active compounds in QLCs by gas chromatography–olfactometry

To identify the volatile compounds responsible for QLC aroma, QLC-Y and QLC-G samples were analyzed by gas chromatography–olfactometry–mass spectrometry (GC-O-MS). Twenty volatile compounds were identified (Table 2), including linalool, nonanal, octanal, sulcatone, carvacrol, β-ionone, and 2-pentylfuran. Nine volatile compounds exhibited high aroma intensity (AI ≥2), such as linalool (AI: 3.0–3.5), nonanal (AI: 3.0–3.5), sulcatone (AI: 0–3.0), and (*E*,*E*)-2,4-heptadienal (AI: 0–2.5). These were considered primary contributors to aroma changes after yellowing treatment.

Four volatile compounds—linalool, nonanal, carvacrol, and β-ionone—were directly perceived as pleasant in sensory evaluation (Table 1). Previous studies have shown that linalool is a key contributor to the sensory profile of high-grade Dianhong black tea (Ma, Gao, Hu et al., 2022), with floral and citrus-like notes and the highest AI among alcohols (Gan et al., 2024). Linalool synthase catalyzes the formation of linalool from geranyl pyrophosphate (Fan et al., 2016; Y. Li et al., 2024). Although these studies reported a decrease in linalool content after yellowing treatment, the relatively slow yellowing process (compared with green tea production) may account for the higher linalool AI scores observed in QLC-Y.

**Table 1:** Differentially abundant volatile compounds (VCs) with ROVA (scores >1).^a^

NO.	Compound	Aroma description^a-d^	RI value⁎	ODT(μg/L)^a-e^	ROVA		Significance
					QLC-Y	QLC-G	
1	(*Z*)-linalool oxide (*pyranoid*)	Sweet, floral, creamy	1174	190.00	1.01 ± 0.05	0.74 ± 0.07	*P* < 0.05
2	(*E*)-linalool oxide (*furanoid*)	Sweet, floral, creamy	1087	190.00	1.69 ± 0.10	1.41 ± 0.31	*P* > 0.05
3	Hexanal	Green	799	4.50	7.00 ± 0.10	3.76 ± 0.77	*P* < 0. 05
4	Nonanal	Sweet	1102	1.00	98.14 ± 6.64	58.71 ± 5.82	*P* < 0. 05
5	Methyl salicylate	Minty, fresh, sweet	1193	40.00	1.45 ± 0.11	0.97 ± 0.11	*P* < 0. 05
6	Heptanal	Chestnut-like, sweet, grass	900	10.00	10.58 ± 0.37	12.45 ± 1.07	*P* > 0. 05
7	Octanal	Fruity	1001	0.59	143.84 ± 9.49	70.51 ± 1.95	*P* < 0. 05
8	(*E*,*E*)-2,4-Heptadienal	Fatty, nutty, hay, green, oily	1009	10,000.00	31.37 ± 1.73	44.2 ± 1.43	*P* < 0. 05
9	Benzeneacetaldehyde	C^**lean, rose-like, floral, and chocolate-like**^	1044	750.89	12.66 ± 0.22	12.93 ± 1.06	*P* > 0. 05
10	*β*-Myrcene	Woody, resinous, musty	988	15.00	–	15.18 ± 1.81	*P* < 0. 05
11	(*E*)-β-ocimene	Warm, foral, herbal, sweet	1045	0.02	–	3.28 ± 0.49	*P* < 0. 05
12	*γ*-Terpinene	Citrus, lemon-like, woody, spicy, juicy	1059	1000	0.98 ± 0.05	3.41 ± 0.29	*P* < 0. 05
13	Sulcatone	Citrus, green	982	68.00	12.00 ± 0.53	29.57 ± 0.08	*P* < 0. 05
14	*β*-Ionone	Violet-like, floral, and raspberry-like	1478	0.01	490.33 ± 7.02	447.33 ± 7.02	*P* < 0. 05
15	(*E*)-linalool oxide (*furanoid*)	Sweet, floral, creamy	1087	190.00	1.69 ± 0.10	1.41 ± 0.31	*p* > 0.05
16	(*E*)-2-Octenal	Fatty	1056	3.00	410.33 ± 21.22	505.67 ± 28.92	*P* < 0. 05
17	Terpinen-4-ol	Pleasant, foral	1183	330.00	0.65 ± 0.04	3.03 ± 0.09	*P* < 0. 05
18	Linalool	Sweet and floral	1098	0.22	350.36 ± 5.77	649.19 ± 5.39	*P* < 0. 05
19	*L*-α-Terpineol	Pleasant, foral	1196	330.00	3.11 ± 0.09	11.3 ± 0.59	*P* < 0. 05
20	Geraniol	Rose-like, sweet, honey-like	1246	7.50	55.38 ± 5.53	114.98 ± 17.66	*P* < 0. 05
21	2-pentyl-Furan	Burnt, sweet, bready, caramel-like	989	4.50	8.77 ± 0.37	10.85 ± 0.69	*P* < 0. 05

a As stated in http://www.thegoodscentscompany.com/search3.php, ^b^(Wang, Qu, et al., 2022), ^c^(Guo et al., 2021), ^d^(Ma, Gao, Linqi, et al., 2022). ^e^(Yue et al., 2023).

⁎ RIs were calculated from published retention time of each compound using a matching score threshold of 780 for the mass spectra in NIST 20 database.

Other alcohols, such as nerol (clean, floral), have been identified as key contributors to chestnut-like aroma (Zhu et al., 2018), and are derived from the degradation of limonene and linalool (Liu et al., 2022). Nerol AI values increased during yellowing (Huang et al., 2023). In contrast, α-terpineol (woody, floral) (Dai et al., 2019), a major aroma constituent of Qianlin green tea (Zhang, Zhang, et al., 2024), was not detected in QLC-Y by GC-O-MS, suggesting that QLC-G retains more woody and floral character.

Saturated fatty aldehydes such as hexanal, heptanal, octanal, nonanal, and phenylacetaldehyde impart grassy, green, fruity, sweet aromas, and most increased in QLC-Y (Table 1). The unsaturated aldehyde *(E,E)-2,4-heptadienal* (AI: 2.5), associated with fatty and mellow notes in black tea (Liu et al., 2022; Yan et al., 2024), was absent in QLC-Y, possibly due to degradation during yellowing. Conversely, β-cyclocitral increased, likely via amino acid degradation or unsaturated fatty acid oxidation under yellowing conditions (Li et al., 2019). (2*E*)-2-Nonenal and (2*E*)-2-Nonenal (AI: 1.0–1.5 each) also increased, enhancing green and sweet aroma intensities—potentially contributing to the more pronounced floral profile in QLC-Y.Among ketones, sulcatone was the most prominent in QLC-G (AI: 3.0) and is known to contribute to oolong and Qingzhuan tea aroma (Xue et al., 2023; Yin et al., 2023). Yellowing appeared to degrade sulcatone, reducing green odor intensity in QLC-Y. In contrast, β-ionone (AI: 1.5–2.0), a major floral enhancer, increased with optimized yellowing (Wei et al., 2023), underscoring the process's role in boosting floral aroma quality.

### 3.8 **Absolute quantification of key** volatile compounds **and aroma recombination in the QLC-Y**

**Fig. 6:**
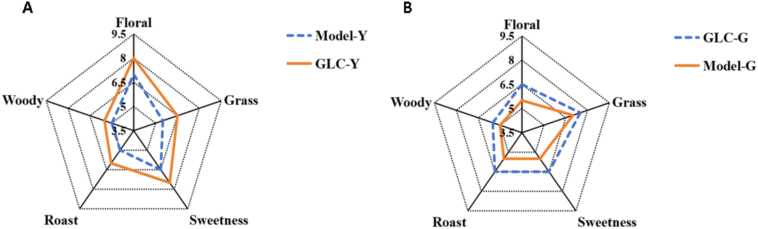
Aroma recombination analysis of key floral compounds.

As discussed earlier, the four odorants with aroma intensities (AI) ≥ 2.5 in QLC were linalool, nonanal, sulcatone, and *(E,E)*-2,4-heptadienal, to further confirm those aroma were the predominant compounds, absolute quantification using the external standard method was conducted to measure their contents in QLC (Table 3). Then, based on the standard curve and ROAV (Table 2), Using these four key floral compounds, the contents of the four odor substances were calculated, and the GLC-Y model (Model-Y) and GLC-G model (Model-G) were established, and which were compared with the original QLC samples by the sensory evaluation. The results showed that the Model-Y were similar to those of the original QLC-Y, and the same as the Model-G. Therefore, both linalool and nonanal were key floral aroma-forming compounds in QLC-Y (Fig. 6).

**Table 2:** Odor descriptions and aroma intensities of volatile compounds in QLC-G and QLC-Y infusions.

No.	Volatile Compounds	RI value^a^	Odor descriptions	Aroma intensity (Mean ± SD)^b^		Significance
				QLC-Y	QLC-G	
1	Linalool	1098	Floral	3.5 ± 0.1	3.0 ± 0.1	*P*<0. 05
2	Nonanal	1102	Sweet	3.5 ± 0.1	3.0 ± 0.1	*P*<0. 05
3	2-Pentylfuran	989	Baking, bean	2.0 ± 0.1	1.5 ± 0.1	*P*<0. 05
4	Octanal	1001	Soap, fruity	2.0 ± 0.1	–	*P*<0. 05
5	Carvacrol	1219	Sweet	2.0 ± 0.2	0.5 ± 0.1	*P*<0. 05
6	β-Ionone	1478	Violet-like, raspberry, floral	2.0 ± 0.2	1.5 ± 0.3	*P*>0. 05
7	Heptanal	900	Soap, fruity	1.6 ± 0.2	1.3 ± 0.2	*P*>0. 05
8	Hexanal	799	Green, grassy	1.5 ± 0.2	1.2 ± 0.2	*P*>0. 05
9	Phenylacetaldehyde	1044	Sweet	1.5 ± 0.2	1.5 ± 0.3	*P*>0. 05
10	(*E*)-oct-2-enal	1056	Green	1.5 ± 0.2	1.5 ± 0.3	*P*>0. 05
11	Linalool OxideI(*Furanoid*)	1087	Woody, floral	1.5 ± 0.2	1.0 ± 0.2	*P*>0. 05
12	Linalool OxideII(*Pyranoid*)	1170	Woody, floral	1.0 ± 0.2	1.0 ± 0.2	*P*>0. 05
13	4-Terpineol	1183	Sweet, floral	1.5 ± 0.2	0.5 ± 0.1	*P*<0. 05
14	*trans*-Nonenal	1120	Grassy green	1.5 ± 0.1	1.0 ± 0.2	*P*<0. 05
15	*trans*-2-Nonenal	1025	Floral	1.5 ± 0.1	1.0 ± 0.1	*P*<0. 05
16	Nerol	1557	Sweet natural citrus magnolia	1.5 ± 0.2	1.0 ± 0.2	*P*<0. 05
17	*β*-Cyclocitral	1276	Burnt	1.0 ± 0.1	–	*P*<0. 05
18	*α*-Terpineol	1196	Earthy, stink	–	1.0 ± 0.1	*P*<0. 05
19	(*Z*)-3-Hexen-1-ol	1570	Green, mushroom-like	–	2.0 ± 0.1	*P*<0. 05
20	Sulcatone	982	Strong green, grassy	–	2.5 ± 0.1	*P*<0. 05
21	*(E,E)-2,4-heptadienal*	1009	Fatty, stale	–	2.5 ± 0.1	*P*<0. 05
22	1-Nonanol	1167	Sweet	–	1.5 ± 0.3	*P*<0. 05

a RI values calculated from n-alkanes on a DB-5MS capillary column. ^b^ The aroma intensities were evaluated by GC-O. “-” Not obtained.

**Table 3:** Standard curves and contents of key aroma-active compounds of QLC infusions.

NO.	Compounds	Standard curve	R^2^	Linear range (μg/L)	Concentrations of QLC infusion (μg/L)	
					QLC-Y	QLC-G
1	Linalool	Y = 0.79 × 10^−3^×-2.08	0.9841	2.07–20,654.90	77.08	142.82
2	Nonanal	Y = 0.23×-315.75	0.9924	1.79–17,866.70	98.14	58.71
3	Sulcatone	Y = 0.37× + 0.0038	0.9998	1.31–13,102.80	816.00	2010.76
4	*(E,E)*-2,4-heptadienal	Y = 2.26 × ^2^ + 8.96×-1.97	0.9965	0.79–404.64	313,700	442,000

### 3.9 Possible formation pathways of key odorants in QLC during yellowing process

The mechanisms underlying the enhanced sweet and floral aroma of QLC-Y were investigated. Previous studies have identified linalool as imparting floral and clean notes (Ye et al., 2025), while nonanal contributes a sweet aroma derived from oleic acid via enzymatic oxidation. Sulcatone, formed through oxidation and dehydrogenation of lavender aldehyde, imparts strong green and grassy odors, whereas *(E,E)*-2,4-heptadienal—associated with fatty, stale-like notes—appears to contribute to the medicinal aroma characteristic of QLC-G. Our results indicate that yellowing enhances the sweet and floral qualities of QLC-Y while diminishing the grassy and stale notes found in QLC-G.

**Fig. 7:**
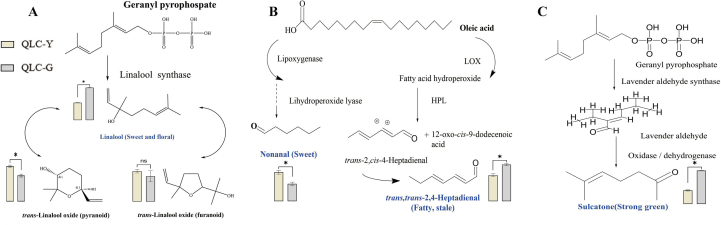
Pathways for the molecular formation of key characteristic QLC aroma compounds. (A) Linalool. (B) Nonanal, *(E,E)-*2,4-heptadienal. (C) Sulcatone.

As discussed earlier, the four odorants with aroma intensities (AI) > 2.5 in QLC were linalool, nonanal, sulcatone, and *(E,E)-2,4-heptadienal*. We therefore propose the molecular formation pathways of these characteristic volatile compounds (Fig. 7). Linalool originates from geranyl pyrophosphate via the catalytic action of linalool synthase (Xu et al., 2024). Nonanal is generated from oleic acid through enzymatic oxidation (Hu et al., 2022). we speculated that during yellowing, gradual exterior-to-interior heat transfer promotes protein degradation and secondary biochemical reactions, which likely increase the concentrations of linalool oxide I (*furanoid*), linalool oxide II (*pyranoid*) and nonanal, thereby intensifying floral and sweet notes. Sulcatone, originating from the oxidative dehydrogenation of lavender aldehyde, is responsible for strong green tones, while *(E,E)-2,4-heptadienal* contributes fatty, stale-like nuances. The yellowing process acts to moderate the contribution of compounds including linalool, (*E*,*E*)-2,4-heptadienal, and sulcatone, thereby promoting the overall aroma balance toward the more desirable sweet-floral character seen in QLC-Y.

Changes in the quantities of key odorants thus reveal the specific metabolic re-routing that takes place during yellowing.(Fig. 7). Most instructively, the decrease in linalool concurrent with the rise in linalool oxides I and linalool oxides II provides direct evidence for the activation of a monoterpene oxidative transformation pathway. Simultaneously, the marked increase in nonanal confirms the sustained activation of the fatty acid oxidation (LOX) pathway, contributing fresh aldehydic notes. Conversely, the reduction in *(E,E)-*2,4-heptadienal may indicate its further oxidation or a divergence in lipid peroxidation routes under these specific conditions. Therefore, the yellowing of QLC appears to be defined by two predominant chemical mechanisms: 1) the oxidative conversion of pre-formed monoterpenes, 2) the continued generation of fatty acid-derived aldehydes. This pathway-specific interpretation offers a clearer mechanistic understanding of the unique flavor formation in *Camellia cuspidata*.

## 4 Conclusion

This study suggests that yellowing treatment may modify the composition of physicochemical and aroma compounds, potentially leading to an enhancement in floral and sweet flavors, along with a reduction in grassy and roasted notes. ROAV and GC-O analyses jointly identified linalool, nonanal, sulcatone, and (*E*,*E*)-2,4-heptadienal as the primary contributors to the floral and sweet flavor notes. Together, these findings establish the chemical basis for the floral aroma of QLC and provide a theoretical foundation for refining processing techniques and guiding the development of new QLC products. Our subsequent research will focus on the precision modulation of processing techniques for Qianlin yellow tea and the targeted development of floral-aroma products, aiming to advance its production from empirical practices toward distinctive and functional tea processing. This will provide technical support for the high-value utilization of specialty agricultural products.

## CRediT authorship contribution statement

**Fei Ye:** Writing – original draft, Methodology, Investigation, Formal analysis. **Xueping Wang:** Writing – original draft, Methodology, Investigation, Formal analysis. **Xiaoyan Qiao:** Writing – original draft, Methodology, Investigation, Formal analysis. **Anhui Gui:** Writing – original draft, Methodology, Formal analysis. **Panpan Liu:** Writing – original draft, Methodology, Formal analysis. **Lin Feng:** Resources, Methodology, Investigation. **Jin Teng:** Resources, Methodology, Investigation. **Jinjin Xue:** Resources, Methodology, Investigation. **Binghua Zhang:** Methodology, Investigation. **Pengcheng Zheng:** Writing – review & editing, Visualization, Resources, Methodology, Conceptualization. **Shiwei Gao:** Writing – review & editing, Visualization, Resources, Methodology, Conceptualization.

## Ethical statement

Sensory evaluations conducted in this study were performed in accordance with relevant institutional guidelines. All panelists were informed about the purpose and procedures of the sensory analysis and provided written informed consent. No personal information of the panelists is disclosed in this manuscript to protect privacy and comply with legal standards. This study did not involve any hazardous materials or unethical procedures.

## Funding sources

This work was supported by the national key R&D program of China (2024YFD1702003 and 2022YFD1600804), the Agriculture Research System of China of MOF and MARA (CARS-19), the Agricultural Science and Technology Innovation Center of Hubei Province (2025–620–000-001-020). Rural revitalization science and technology project of Hubei province (2023BBB142 and 2023BBB071).

## Declaration of competing interest

The authors declare that they have no known competing financial interests or personal relationships that could have appeared to influence the work reported in this paper.

## Data Availability

Data will be made available on request.

## Appendix A Supplementary data

Supplementary material: The supplementary data is accessible in the online version.
