# Supplementary material for: Yellowing treatment transforms sensory profile of Qianlin cha (Camellia cuspidata): Key aroma compounds and quality enhancement
Source: Food Chem X. 2026 Jun 18;37:104102. doi: 10.1016/j.fochx.2026.104102 (PMC13324664; doi:10.1016/j.fochx.2026.104102)
Supplement: Supplementary file 1 — Supplementary material: The supplementary data is accessible in the online version. [file mmc1.docx]

**Supplementary material**

**Table 1.** The ROAV values of main aroma components in QLC-G and QLC-Y

| **Compound** | **GLC-G** | **GLC-G** | **GLC-G** | **GLC-Y** | **GLC-Y** | **GLC-Y** |
| --- | --- | --- | --- | --- | --- | --- |
| (*E*)-3-Hexen-1-ol | 174.80 | 168.00 | 168.40 | 370.00 | 389.60 | 394.00 |
| (*Z*)-linalool oxide (*pyranoid*) | 0.00 | 0.00 | 0.00 | 10.50 | 10.31 | 11.64 |
| Phenylethyl Alcohol | 0.00 | 0.00 | 0.00 | 104.50 | 100.00 | 115.00 |
| Terpinen-4-ol | 12.94 | 12.22 | 12.36 | 2.68 | 2.85 | 2.53 |
| Linalool | 654.90 | 648.47 | 644.20 | 346.77 | 347.28 | 357.02 |
| L-*α*-Terpineol | 11.06 | 11.97 | 10.86 | 3.04 | 3.08 | 3.21 |
| (*E*)-linalool oxide (*furanoid*) | 166571.43 | 186857.14 | 250142.86 | 245857.14 | 225571.43 | 254142.86 |
| Geraniol | 128.27 | 94.93 | 121.73 | 50.67 | 54.00 | 61.47 |
| 2-pentyl-Furan | 6.75 | 6.50 | 5.95 | 5.40 | 5.16 | 4.96 |
| Hexanal | 3.37 | 3.27 | 4.65 | 6.92 | 6.96 | 7.11 |
| Heptanal | 13.66 | 9.05 | 11.63 | 12.00 | 9.37 | 12.36 |
| Octanal | 11300.00 | 10625.00 | 9900.00 | 5037.50 | 5250.00 | 5312.50 |
| (*E*,*E*)-2,4-Heptadienal | 10712.50 | 11012.50 | 11425.00 | 7637.50 | 7550.00 | 8337.50 |
| Benzeneacetaldehyde | 197.20 | 196.80 | 226.40 | 198.40 | 204.80 | 204.40 |
| (*E*)-2-Octenal | 537.00 | 500.00 | 480.00 | 434.00 | 393.00 | 404.00 |
| Nonanal | 57.81 | 53.40 | 64.93 | 99.70 | 90.85 | 103.86 |
| Thymol | 0.00 | 0.00 | 0.00 | 2.38 | 2.69 | 2.46 |
| *o*-Cymene | 13.64 | 12.06 | 14.20 | 5.71 | 5.83 | 5.92 |
| *β*-Myrcene | 14.06 | 14.20 | 17.26 | 0.00 | 0.00 | 0.00 |
| (*E*)-β-ocimene | 12712.50 | 12787.50 | 16325.00 | 0.00 | 0.00 | 0.00 |
| *γ*-Terpinene | 27371.43 | 24328.57 | 28714.29 | 7442.86 | 7500.00 | 8100.00 |
| 6-Methyl-5-Hepten-2-one | 29.50 | 29.65 | 29.55 | 11.40 | 12.40 | 12.20 |
| *trans*-β-Ionone | 448.00 | 420.00 | 594.00 | 397.00 | 483.00 | 491.00 |
| Methyl salicylate | 803.33 | 726.67 | 901.67 | 1105.00 | 1255.00 | 1261.67 |
| Hexanoic acid, 3-hexenyl ester | 32.25 | 26.50 | 21.17 | 18.42 | 18.83 | 22.58 |
| n-Valeric acid cis-3-hexenyl ester | 71.00 | 59.00 | 70.60 | 83.40 | 92.40 | 92.80 |

**Table 2.** The aroma attributes of QLC-G and QLC-Y

| **Aroma attributes** | **GLC-G** | **GLC-G** | **GLC-G** | **GLC-Y** | **GLC-Y** | **GLC-Y** |
| --- | --- | --- | --- | --- | --- | --- |
| Floral | 6.50 | 6.60 | 6.40 | 8.00 | 8.10 | 7.90 |
| Grass | 7.50 | 7.65 | 7.35 | 6.5 | 6.44 | 6.57 |
| Sweetness | 6.50 | 6.63 | 6.37 | 7.500 | 7.43 | 7.58 |
| Roast | 6.50 | 6.63 | 6.37 | 6.00 | 5.94 | 6.06 |
| Woody | 5.50 | 5.61 | 5.39 | 5.50 | 5.445 | 5.56 |
